# Oxytocin Dynamics in the Body and Brain Regulated by the Receptor for Advanced Glycation End-Products, CD38, CD157, and Nicotinamide Riboside

**DOI:** 10.3389/fnins.2022.858070

**Published:** 2022-07-07

**Authors:** Haruhiro Higashida, Kazumi Furuhara, Olga Lopatina, Maria Gerasimenko, Osamu Hori, Tsuyoshi Hattori, Yasuhiko Hayashi, Stanislav M. Cherepanov, Anna A. Shabalova, Alla B. Salmina, Kana Minami, Teruko Yuhi, Chiharu Tsuji, PinYue Fu, Zhongyu Liu, Shuxin Luo, Anpei Zhang, Shigeru Yokoyama, Satoshi Shuto, Mizuki Watanabe, Koichi Fujiwara, Sei-ichi Munesue, Ai Harashima, Yasuhiko Yamamoto

**Affiliations:** ^1^Department of Basic Research on Social Recognition and Memory, Research Center for Child Mental Development, Kanazawa University, Kanazawa, Japan; ^2^Laboratory of Social Brain Study, Research Institute of Molecular Medicine and Pathobiochemistry, Krasnoyarsk State Medical University named after Professor V.F. Voino-Yasenetsky, Krasnoyarsk, Russia; ^3^Department of Neuroanatomy, Kanazawa University Graduate School of Medical Sciences, Kanazawa, Japan; ^4^Department of Neurosurgery, Kanazawa Medical University, Kanazawa, Japan; ^5^Faculty of Pharmaceutical Sciences, Center for Research and Education on Drug Discovery, Hokkaido University, Sapporo, Japan; ^6^Department of Biochemistry and Molecular Vascular Biology, Kanazawa University Graduate School of Medical Sciences, Kanazawa, Japan

**Keywords:** oxytocin, RAGE, transport, endothelial cells, CD38

## Abstract

Investigating the neurocircuit and synaptic sites of action of oxytocin (OT) in the brain is critical to the role of OT in social memory and behavior. To the same degree, it is important to understand how OT is transported to the brain from the peripheral circulation. To date, of these, many studies provide evidence that CD38, CD157, and receptor for advanced glycation end-products (RAGE) *act as* regulators of OT concentrations in the brain and blood. It has been shown that RAGE facilitates the uptake of OT in mother’s milk from the digestive tract to the cell surface of intestinal epithelial cells to the body fluid and subsequently into circulation in male mice. RAGE has been shown to recruit circulatory OT into the brain from blood at the endothelial cell surface of neurovascular units. Therefore, it can be said that extracellular OT concentrations in the brain (hypothalamus) could be determined by the transport of OT by RAGE from the circulation and release of OT from oxytocinergic neurons by CD38 and CD157 in mice. In addition, it has recently been found that gavage application of a precursor of nicotinamide adenine dinucleotide, nicotinamide riboside, for 12 days can increase brain OT in mice. Here, we review the evaluation of the new concept that RAGE is involved in the regulation of OT dynamics at the interface between the brain, blood, and intestine in the living body, mainly by summarizing our recent results due to the limited number of publications on related topics. And we also review other possible routes of OT recruitment to the brain.

## Introduction

It has been established that OT is not only secreted from the posterior pituitary into the circulation but is also released in the brain, where it has very diverse behavioral effects (Russell et al., 2003; Leng et al., 2015; Higashida, 2016; Russell, 2018). Hypothalamic neurons secrete oxytocin (OT) into the brain from dendrites and axons to function as an endogenous factor in species-specific social memory and behaviors, including emotion, interaction, and bonds (Baumgartner et al., 2008; Bridges, 2015; Huang et al., 2015; Numan and Young, 2016; Feldman, 2017; Higashida et al., 2018, 2019a,b; Jurek and Neumann, 2018; Borowiak and von Kriegstein, 2020; Carter et al., 2020; Quintana and Guastella, 2020; Tolomeo et al., 2020; Froemke and Young, 2021; Grinevich and Neumann, 2021; Yeomans et al., 2021; Zheng and Kendrick, 2021; Carter, 2022). CD38 and CD157 play an essential role in central OT release (Jin et al., 2007; Higashida et al., 2017b). The two molecules are related cell-surface antigens that produce cyclic ADP-ribose (cADPR), a calcium-mobilizing second messenger, from the substrate nicotinamide adenine dinucleotide (NAD; Lee and Zhao, 2019; Chini et al., 2020, 2021). cADPR functions as the potential intracellular second messenger that triggers Ca^2+^ mobilization from ryanodine receptor Ca^2+^ pools to produce cellular responses (Lee and Zhao, 2019; Avraham et al., 2021; Okamoto and Takasawa, 2021). In the hypothalamus, cADPR activates the elevation of intracellular free Ca^2+^ concentrations and subsequently elicits Ca^2+^-dependent OT release from oxytocinergic neurons (Jin et al., 2007; Higashida et al., 2011). When this signaling pathway is prevented in CD38 knockout (CD38KO) mice, social memory and recognition or parental nurturing behavior are disrupted mainly owing to reduced OT release (Jin et al., 2007). Localized re-expression of human CD38 in the hypothalamus or simple subcutaneous administration of OT in CD38KO mice confirms the importance of CD38 and OT in social behavior (Chong et al., 2017). The phenotypes of CD157KO and CD38KO mice in social behavior are partly shared, but the significant differences are anxiety- and depression-like behaviors. In addition, CD157KO mice showed social avoidance as a substantial impairment.

**FIGURE 1:**
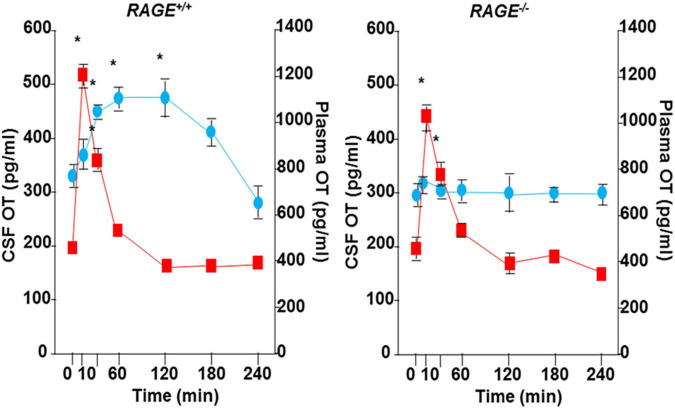
Transportation of peripherally administrated oxytocin (OT) to the brain. Concentrations of OT in plasma (red) and cerebral spinal fluid (CSF) of the cisterna magna (blue) after subcutaneous injection of 30 ng OT (100 ng/mL × 0.3 mL) in wild-type (*RAGE*^+/+^) or RAGE KO (*RAGE*^–/–^) mice (*n* = 3–16/data point, *P < 0.05 at time 0). The data shown are modified from Figure 4 of Yamamoto et al. (2019). OT was measured in un-extracted samples.

A single nasal administration of OT, as an exogenous factor, in healthy humans showed increased social attention and interaction (Veening and Olivier, 2013; Quintana et al., 2018; Tanaka et al., 2018; Kurokawa et al., 2021, 2020) and in subjects with social behavioral deficits who suffer from autism spectrum disorders and schizophrenia (Munesue et al., 2016, 2010; Okamoto et al., 2016; Quintana et al., 2017; Higashida et al., 2019b; Huang et al., 2021). During this process, peripheral OT is assumed to reach the brain *via* the nasal-to-brain or blood-to-brain routes (Quintana et al., 2018, 2017; Lee and Jayant, 2019; Martins et al., 2020; Grinevich and Neumann, 2021; Yeomans et al., 2021). Plasma OT, however, is assumed to not cross the blood-brain barrier (BBB) because hydrophilic OT is larger than 1,000 Da in its molecular weight (Avraham et al., 2021). However, there was evidence that OT can pass through the BBB in rodent studies (Jin et al., 2007; Neumann et al., 2013), in that after subcutaneous and intraperitoneal injection of OT, OT concentrations in the cerebrospinal fluid (CSF) increase (Jin et al., 2007; Yamamoto et al., 2019), as shown in Figure 1. In humans, along with the same analogy, it could be speculated that nasal OT permeates into the bloodstream through micro blood vessels, which are very well developed in the nasal mucosal membranes (Beju et al., 2004). OT can subsequently be taken up by the brain from the circulation.

To make the above estimation of brain recruitment of OT through the BBB, molecular mechanisms of entry were strongly expected for a decade. Higashida et al. (2017a) published the first evidence in 2017 that the receptor for advanced glycation end-products (RAGE) is a transporter of OT from the intestinal lumen to the body fluid at the intestinal barrier in male mice. Subsequently, the binding and transport properties of RAGE at the BBB have been shown in 2019 to 2021 by Yamamoto and Higashida (2020), and our other colleagues (Shimizu et al., 2020; Gerasimenko et al., 2021; Leerach et al., 2021; Munesue et al., 2021). Biochemically, the binding between OT and RAGE was demonstrated by the plasmon resonance method (Yamamoto et al., 2019). The transport of OT by RAGE after binding was demonstrated in the *in vitro* BBB system in two chambers, which represent the luminal (blood) and abluminal (brain) sides separated by cultured endothelial cells, astrocytes, and pericytes (Yamamoto et al., 2019). In such a system, RAGE functions as a transporter of OT primarily from the blood to the brain, with approximately 10-fold greater efficiency than in the reverse direction (Yamamoto et al., 2019; Zheng and Kendrick, 2021). Importantly, these data indicate that this transport is unidirectional from the blood to the brain chambers and is in saturation mode. In addition, we calculated the bioavailability of this transfer to be approximately 0.2% in mice. We compare these values with the values described by others (Table 1). This type of semi-unidirectional transport of OT was mostly disrupted by knockout (KO) of the mouse gene for RAGE, *Ager*, or knockdown of endothelial RAGE expression by siRNA (Yamamoto et al., 2019). The three forms of RAGE are known as follows: One is full-length RAGE, expressed in the membrane of vascular endothelial cells, membrane-bound RAGE (mRAGE; Mackic et al., 1998; Schmidt et al., 2000; Schmidt and Stern, 2001; Stern D. M. et al., 2002; Stern D. et al., 2002). Second, endogenous secretory RAGE, which is the product of an alternatively spliced mRNA that lacks a membrane-spanning domain and is found in the blood circulation (Yonekura et al., 2003; Harashima et al., 2006). Third, the third form is also soluble RAGE, which is an ectodomain-shedding form (Schmidt and Stern, 2001). It has recently been shown that these soluble forms act as a buffer for OT (Munesue et al., 2021). In addition, concerning intranasal administration of OT, although direct and specific routes have been proposed from the nasal cavity to the human brain (amygdala; Martins et al., 2020), when OT was applied to the nasal cavity in RAGE KO mice, no or little increase in OT was observed in the CSF. This may show that the direct nasal brain route, which has not yet been clearly identified, seems to be sensitive to mRAGE.

**TABLE 1:** Estimated bioavailability of exogenous oxytocin into the brain.

	Sex	Drug	Dose	Injection	Min	%Availability	Extraction references/Method	
Rat	Male	OT	5 μg	s.c.	10	0.02	+/RIA	Neumann et al., 2013
Pig	Female	OT	50 μg	i.n.	10	0.001	-/ELISA	Striepens et al., 2013
Rat	Male	OT	500 μg	i.n.	10	2	-/LC/MS	Tanaka et al., 2018
Mouse	Male	OT	30 ng	i.p.	30	0.2	-/EIA	Yamamoto et al., 2019
Mouse	Male	OT*	30 ng	i.p.	30	0.3	+/LC/MS	Yamamoto et al., 2019
Human	Male	OT	50 μg	i.n.	35	0.1	+/RIA	Rault, 2016
Monkey		OT	1 ng	Culture	30	1.2	-/EIA	Yamamoto et al., 2019
Monkey		OT	10 ng	Culture	30	0.21	-/EIA	Yamamoto et al., 2019

*Oxytocin isoleucine [^13^C, ^15^N] OT, as described in Higashida et al. (2017a).

*RIA, radio immunoassay; ELISA, enzyme-linked immunosorbent assay; LC/MS, liquid chromatography mass spectroscopy; EIA, enzyme immunoassay; s.c., subcutaneous; i.n. intranasal; i.p., intraperitoneal; and i.v., intravenous.*

Originally, functional roles of advanced glycation end-products (AGEs) and their receptors (RAGE) have been reported in the pathological mechanism (secondary damages) underlying diabetic complications, such as diabetic cardiomyopathy, retinopathy, nephropathy, and intestinal hemorrhage (Yonekura et al., 2005, 2003; Raman et al., 2006; Yamamoto et al., 2007; Manigrasso et al., 2018). An example is that the expression of AGE and RAGE in the small intestine has been reported in healthy animals, and their expression levels are upregulated in diabetic rodents (Harashima et al., 2006; Hu et al., 2021). Increased expression of AGE and RAGE may contribute to diabetic tissue dysfunction. Therefore, based on the vast number of disease-related publications, it is challenging to speculate productively about the transport function of OT, a peptide hormone.

RAGE is typically involved in remodeling vascular endothelial cells of the retina and kidney as a secondary injury in diabetic pathological conditions (Yamamoto et al., 2001). AGEs are a broad heterogeneous group of compounds formed by non-enzymatic reactions. The accumulation of endogenous and exogenous AGEs has been implicated in the pathogenesis of numerous diseases in humans, such as diabetes (Schmidt et al., 2000; Stern D. M. et al., 2002; Stern D. et al., 2002). RAGE has been studied mainly related to lifestyle illnesses such as metabolic diseases, diabetes, hypertension, and secondary damage to various organs, including blood vessels (Yonekura et al., 2005). In the neuronal degeneration processes of Alzheimer’s disease, RAGE plays a pivotal role in the efflux of amyloid-beta protein from the brain (Mackic et al., 1998). In addition, RAGE has been shown to relate to psychiatric diseases. RAGE- or S100B-involvement in schizophrenia has been reported as a biomarker (Kouidrat et al., 2015). Therefore, not only the transport impairment of OT to the brain but also RAGE levels seem to be associated with ASD (Yamamoto and Higashida, 2020).

Our recent findings on RAGE suggest that RAGE has a new physiological role in the body, especially in the transport of OT, which has not been shown in the RAGE research field. Therefore, it is rational to review the new concept of RAGE- or nicotinamide riboside-dependent regulation of OT transport from the perspective of the dynamics of OT in the body. However, because one limitation is the limited number of articles intensively focused on this topic, to our knowledge, it is necessary to cite papers published by the laboratories of the current authors. Furthermore, we will discuss changes in brain OT concentrations after gavage supplementation of one of NAD precursors in mice, nicotinamide riboside. This precursor is used in the synthesis of NAD in the salvage pathway.

NAD is synthesized by a salvage pathway of the vitamin precursors, including nicotinic acid, nicotinamide mononucleotide, and nicotinamide riboside, or from tryptophan in the *de novo* pathway (Nikiforov et al., 2015). Thus, the exogenous administration of nicotinamide riboside can increase the synthesis of NAD (Braidy et al., 2019). Concerning the elevation of mouse liver NAD, it is shown that nicotinamide riboside is more available orally than nicotinamide mononucleotide and nicotinic acid. This finding validates nicotinamide riboside’s role as a favored NAD precursor (Trammell et al., 2016). Like nicotinic acid and nicotinamide mononucleotide, nicotinamide riboside is a natural product found in milk. It is incorporated into the intracellular NAD pool. Nicotinamide riboside could be used as a general supplement for people who have adverse reactions to nicotinic acid or nicotinamide mononucleotide (Trammell et al., 2016). In brain tissue activity, NAD synthetase is dramatically low, which makes nicotinic acid unsuitable as a supplement in this case (Mori et al., 2014). Nicotinamide riboside has already been used as a supplement or therapeutic agent to elevate or maintain cellular NAD contents (Ear et al., 2019). Recently, it has been shown that NAD is consumed more *via* an increase of CD38 in aged subjects. Thus, inhibition of CD38 or an increase in NAD may lead to a longer life span (Tarragó et al., 2018; Chini et al., 2021), which is another intriguing topic.

## Oxytocin Dynamics in the Intestine

### Oxytocin in Breast Milk

One of OT sources to babies is breastmilk (Takeda et al., 1986; Muranishi et al., 2016; Stevenson et al., 2020). The milk is produced from the mother’s general circulation. In our preliminary study of breast milk, OT was approximately 70–120 pg/ml in three lactating human mothers [postpartum days (PPDs) 1–5; Yamamoto and Higashida, unpublished data]. Our values are higher than those of a previous study [approximately 10 pg/ml, (Takeda et al., 1986)], probably due to the different methods used.

The transfer of OT from circulation to milk is regulated by the so-called blood-milk barrier (Nielsen et al., 2020; Strasser et al., 2021; Wellnitz and Bruckmaier, 2021), which is rather leaky compared to the BBB but retains selectivity, especially in bacteria and immunological molecules in humans (Nielsen et al., 2020). The permeability of OT changes under different conditions in dairy cows (Strasser et al., 2021). It would be interesting to examine whether RAGE functions at this blood-milk barrier.

### Absorption of Oxytocin in the Digestive System

In mouse infants, plasma OT originates from the secretion of the posterior lobe of the pituitary gland into the circulation and from the absorption of OT in the mother’s milk into the blood through intestinal permeability (Higashida et al., 2017a). The molecular mechanism underlying the absorption of orally administered OT across the intestinal epithelial barrier in infants revealed that RAGE mediates intestinal OT transmission (Higashida et al., 2017a).

OT with nine amino acids is digested by intestinal peptidases but is relatively stable in gastric acid in humans (Takeda et al., 1986; Roca Rubio et al., 2021). OT in early life plays a role in the development of the social brain and in establishing social behavior in adults in humans and rodents (Higashida et al., 2011; Guastella et al., 2018; Carter et al., 2020). Therefore, breast milk not only feeds beneficial microbes and provides immunity but also appears to aid well-being by improving human communication (Lakkireddy et al., 2016; Krol and Grossmann, 2018; Chastant and Mila, 2019; Froemke and Young, 2021; Noel et al., 2021). OT in breast milk can be absorbed intact from the digestive tract into the blood of human neonates (Takeda et al., 1986). OT absorption is a RAGE-mediated process after the onset of gut closure in mice, as reported by Higashida et al. (2017a).

### Plasma Oxytocin Levels After Oral Delivery in Young and Adult Mice

**FIGURE 2:**
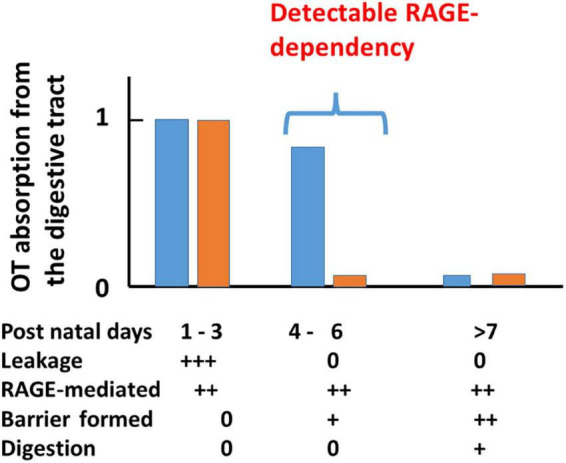
A scheme for plasma OT (OT) concentrations in wild-type (blue) and receptor for advanced glycation end-product (RAGE) knockout (KO; orange) mouse pups after oral OT administration. Plasma OT levels in pups 10 min after oral administration of 1 μg of OT/mouse (10 μL). Blood samples were collected from the hearts of male and female pups on the indicated postnatal days (PNDs). Plasma OT levels are schematically illustrated as bars during three periods after birth. The three time periods (1–3, 4–6, and >7 days) were classified by various factors. During PNDs 1–3, OT levels are equally high in both genotypes, owing to mainly OT leakage from the intestinal mucosa to body fluid where an intestinal barrier has not formed. During PNDs 4–6, in which OT leakage is dramatically decreased because an intestinal barrier has formed. Plasma OT is relatively high in wild-type pups but not in RAGE KO pups because RAGE-dependent OT transport mainly contributes to OT concentrations. During PNDs > 7, in wild-type pups, intestinal barrier and OT cleavage by digestion mask RAGE-dependent OT transport, which resulted in no apparent OT increases. Symbols (0, +, and ++) represent little to no permeability or presence to the highest levels in wild-type or RAGE KO mice. This figure is modified from Figure 3 by Higashida et al. (2017a).

The possibility of transport can be examined with plasma OT levels at 10 min after oral delivery of 10 μL synthetic OT solution (200 ng/pup) and compared with those after the same volume of saline to mouse pups after starvation for 30 min during the development period of suckling (from PND 1 to PND 15; Higashida et al., 2017a). Plasma OT concentrations were markedly higher in neonates to 5-old day pups (PND 1-5) and suddenly dropped at PND 6 in wild-type pups (Figure 2). This finding resembled the results of insulin-like growth factor 1 in suckling piglets (Xu and Wang, 1996). These increases are largely due to leakage from the intestine to body fluid before intestinal barrier formation during PND 1–3 in both genders (Camilleri, 2019). During PND 4–6 when leakage was suppressed by the formation of an intestinal barrier, OT increases are due to RAGE-dependent OT transport from the intestinal epithelium in the wild-type mice. In contrast, there is no increase in RAGE KO mice because of its absence. After PND 7, there was no transport, reflecting little or no transport by an increased barrier and shorter OT due to increased digestive activity (breakdown of OT) masking transport in both genders.

In the case of RAGE KO mice, after oral OT administration, plasma OT concentrations increased from PND 1 to 3. In contrast to the wild-type, increases in OT concentration were not observed after PND 4 (Figure 2). Similar elevated levels of OT on PND 1 to 3 in both genotypes suggest leakage, probably because no barrier was formed. No increase in RAGE KO mice indicates that this uptake between PND 4 and 5 in wild-type mice is RAGE-dependent. Only in this very narrow time window, during which the intestinal barrier seems to have not formed, was the dependency on RAGE revealed (Figure 2).

**FIGURE 3:**
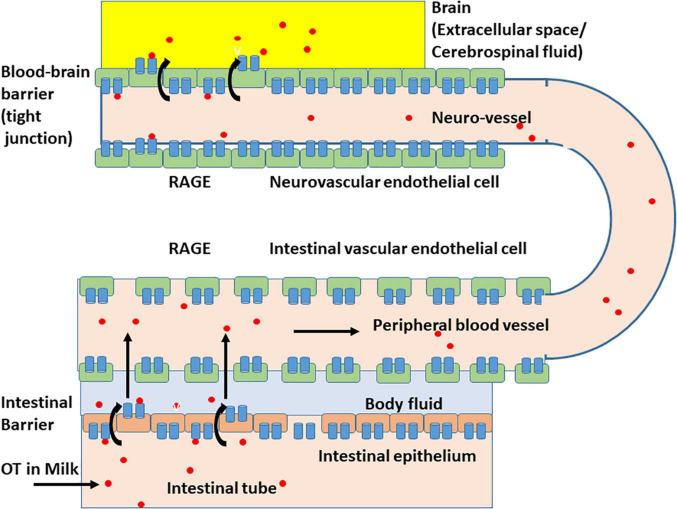
Schematic representation of the oxytocin transport by membrane RAGE (mRAGE) across the intestinal and blood-brain barriers. (Lower) Milk-born or synthetic oxytocin (red circles) in the lumen of the small intestine is transported by mRAGE in intestinal epithelial cells first to body fluid (light blue) and then to blood in (intestinal) blood vessels with no or little barrier. In the lumen of blood vessels, oxytocin (OT; red circles) binds to mRAGE (blue). (Upper) mRAGE and OT interact with each other and internalizes to endothelial cells in small neurovascular vessels at the BBB to the extracellular space or CSF (yellow). The detailed mechanism for transport is not yet known, and OT is likely transported by transcytosis.

The effect of digestion was limited because, when higher doses of OT were applied in adult mice in which digestion appears to be fully active, plasma levels of OT were elevated (Higashida et al., 2017a). These data suggest that milk-born OT can be transmitted across the digestive tract into the blood circulation, including the intestinal mucus and epithelial cells, and OT can cross the intestinal epithelial barrier (Figure 3; Higashida et al., 2017a; Manigrasso et al., 2018). This absorption from the intestine is not strain-specific because identical absorption was observed in the pups of the ICR and C57BL6 strains.

Since then, oral OT supplementation may be advantageous for OT drug development (Higashida et al., 2017a; Yamamoto and Higashida, 2020). A recent study demonstrated that OT administered orally can be transmitted into the blood if it is prevented from degradation in the stomach by pretreating mice with a proton pump inhibitor (Maejima et al., 2020), and using mass spectrometry to evaluate the non-radioactive isoform of OT, we confirmed that OT was absorbed in its intact form. RAGE is abundant in intestinal epithelial cells of villi in both suckling pups and adults in mice (Higashida et al., 2017a) and is an integral component of the intestinal tract.

### Oxytocin Levels in the Hypothalamus After Oral Delivery in Mouse Neonates

**FIGURE 4:**
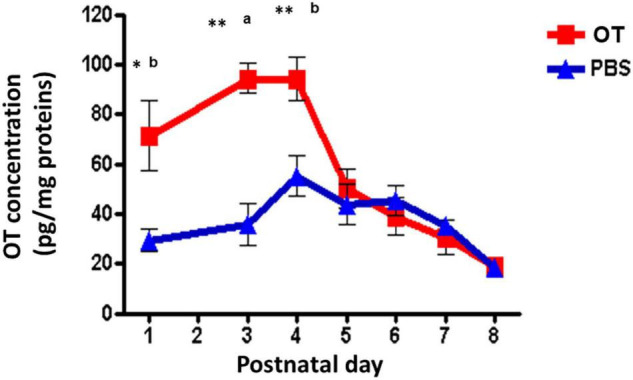
Oxytocin (OT) levels in the hypothalamus of wild-type pups after oral administration of OT. Hypothalami are collected 10 min after oral administration of OT (1 μg in 10 μL) or the same volume of saline (PBS). The tissues were homogenized, and OT was measured by an enzyme immunoassay method. Significant differences between OT and saline at ^a^*P* < 0.001,^b^*P* < 0.01. Significantly different from postnatal day (PND) 6 at *P < 0.01. **P < 0.001. OT levels are significantly higher on PND 1 (*P* < 0.05) and PND 4 (*P* < 001) from PND 5. Experiments were carried out essentially as described in Figure 3 by Higashida et al. (2017a).

One question remains whether orally administered OT in the digestive tract can immediately enter into the brain. Indeed, it is very easy to estimate that OT could be transported by RAGE through two barriers in wild-type mice (Higashida et al., 2017a), RAGE in the intestine intakes OT from the digestive tract and then into the blood circulation, followed by the transport crossover of the BBB to the brain from the blood. Since direct evidence for this was missing, we present our preliminary results on this point. Since it is very difficult to obtain CSF from the small brains of mouse neonates, to assess this transportation, we measured OT levels in homogenates of the mouse hypothalamus after oral application of OT or saline at different PNDs. As shown in Figure 4, the OT levels in hypothalamic tissue were higher in pups treated with 100 μL of OT (10 ng/mouse) than in saline control pups at PNDs 1, 3, and 4 (Lopatina et al., unpublished data). Concentrations reached the same levels as those of mice administered saline at PNDs 5–8. This result strongly suggests that milk-born OT can be absorbed from the digestive tract during suckling and can reach the brain in babies.

### Plasma Oxytocin Levels After Administration of Lipo-oxytocin-1 in Adult Mice

Highly homologous arginine vasopressin has been shown to bind to RAGE but is not transported efficiently and the nine amino acid peptide, bradykinin, does not completely bind to RAGE (Higashida et al., 2017a). These findings suggest that the RAGE-dependent transport system of OT possesses different efficacy compared with different peptides or the recognition ability of structural differences between OT and other peptides, indicating the specificity for OT.

The next question is whether RAGE can transport a lipidated OT analog, lipo-oxytocin-1 (LOT-1), which contains two palmitoyl acid chains [-CO(CH_2_)_14_CH_3_] in cysteine and tyrosine residues (Mizuno et al., 2015; Cherepanov et al., 2017a,b, 2019). LOT-1 is one of our OT analog series and is composed of natural chemicals (Ichinose et al., 2019).

Bioactive peptide hormones have been used for clinical therapy (Davis et al., 2015). However, the therapeutic use of natural peptide hormones is substantially limited due to pharmacokinetic properties, such as poor absorption and brain transport, low metabolic stability, and rapid excretion from the kidney. On the other side, methods have been developed to mitigate the undesired pharmacokinetic properties of natural peptides (Egleton and Davis, 2005). One such methodology for this purpose, especially for the elongation of the lifetime in blood, is lipidation, which involves conjugating a peptide hormone with a long fatty acid (Eskandari et al., 2013).

Although the effect of lipidation of the parent molecule on BBB penetration is less documented, small-sized hydrophobic molecules can penetrate the BBB better than hydrophilic molecules (Eskandari et al., 2013). Thus, it is speculated that the lipidation strategy with increased hydrophobicity can extend its lifetime in the blood and improve its brain delivery capacity. An OT analog, LOT-1, was synthesized based on this hypothesis.

The effect of the new OT analog on social behavior defects was evaluated in CD157 KO mice. CD157 KO mice display psychiatric features of non-motor symptoms of Parkinson’s disease, such as anxiety-related and depression-like behaviors, fear, and social avoidance (Lopatina et al., 2014; Mizuno et al., 2015; Kasai et al., 2017). Furthermore, it has been shown that this social impairment in CD157 KO mice is readily recovered by single peripheral administration of OT (Lopatina et al., 2014; Higashida et al., 2017b), suggesting that these phenotypes may be shared with other psychiatric impairments, for example, autism spectrum disorders and schizophrenia. LOT-1 has an advantage over native OT for long-lasting *in vivo* effects. In contrast, OT has better recovery effects than this analog on social impairment shortly after the treatmen (Mizuno et al., 2015).

We can also biologically examine whether LOT-1 can be cleaved into the OT and side chains in the digestive tract or blood within the measured time window for 240 min. This question is also important to estimate how long lapidated OT takes time for cleavage when we consider LOT-1 as a prodrug of OT as a pill in clinical application in the future.

**FIGURE 5:**
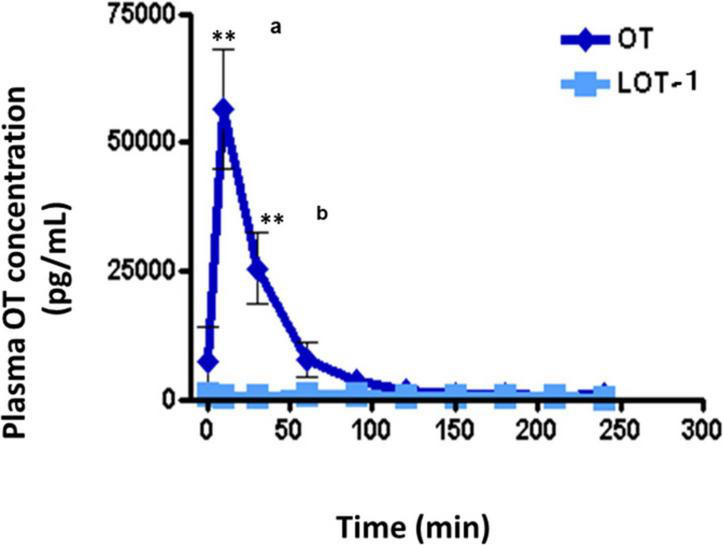
Plasma levels of oxytocin (OT) in adult males after oral administration of lipo-oxytocin 1 (LOT-1). Blood samples were collected from the hearts of adult males after oral administration of OT or LOT-1 (each 10 μg × 100 μL). Significant differences between OT and LOT-1 (^a^*P* < 0.001, ^b^*P* < 0.01). Significantly different from the control value at **P < 0.001. *n* = 3–8. Experiments were performed according to figure by Higashida et al. (2017a).

No increase in plasma OT concentration was observed in wild-type adult male mice after oral administration of LOT-1 (10 μg/mouse) for 240 min, while in identical OT experiments, a substantial increase in plasma OT concentrations was observed after 10 min (Figure 5). OT levels returned after 60 min. As expected, after oral administration of LOT-1 (10 μg/mouse), plasma OT levels did not increase in RAGE KO mice (data not shown). These results suggest that LOT-1 did not cleave to OT within 240 min in the digestive tract or did not absorb LOT-1 due to structural selectivity in RAGE recognition in mice. Furthermore, if cleaved, the increase could be detected as an OT. Alternatively, even if RAGE can recognize LOT-1 and transport it, LOT-1 did not break down to OT in the blood in 240 min.

Unfortunately, this experiment was not sophisticated. The most significant limitation of this type of experiment is the lack of measurement of LOT-1 by mass spectroscopy. However, we wanted to know how and when OT breakdown occurred during 4 h in the commonly used biological system; if so, this information allows us to consider LOT-1 as a prodrug (Cherepanov et al., 2017b). In addition, although indirect, RAGE’s recognition capacity or sites should be evaluated in the near future.

## Receptor for Advanced Glycation End-Products-Dependent Oxytocin Transport to the Brain

### Receptor for Advanced Glycation End-Products in Vascular Units in Mice

Immunohistochemically, RAGE in neurovascular patterns is limited to endothelial cells recognized by co-localization with CD31, a marker of vascular endothelial cells in the brain (Noguchi et al., 2020). RAGE is probably located in coated pits, showing an association with caveolin in the wild-type (Yamamoto et al., 2019). CD31 is located on the endothelial cells’ luminal and abluminal plasma membranes. CD31 immunofluorescence intensity on the luminal surface is more abundant than on the abluminal surface (Mori et al., 2006). Usually, in doubly immunostained cryosections, anti-CAV-1α antibodies are shown to be small and individual but punctate fluorescent structures (caveolae), primarily associated with the endothelial cell surface anti-CD31 antibody. Detecting their co-localization is a vital observation to investigate endocytosis of the RAGE-OT complex at the coated pit (Ramirez et al., 2002).

A preliminary study on Z-stack images confirmed RAGE and CD31 co-localization (Supplementary Figure 1), indicating that RAGE resides within mouse vascular endothelial cells and the RAGE action sites are on endothelial cells, alternatively on the BBB (Yamamoto et al., 2019).

### Immunostaining of Human Vascular Units

Using a piece of brain tissue from adult human patients with glioma, we stained them with a RAGE antibody. Immune precipitation was observed on the endothelial cell surface of human brain tissue (Supplementary Figure 2). The presence of RAGE in the neurovascular unit of humans strongly supports the RAGE-dependent OT transport system in the human BBB (Figure 3).

### Other Routes of the Brain Transport of Oxytocin

There find reports in which OT concentrations in the various brain regions and/or CSF are increased after peripheral administration in different mammals. In human studies, changes in images at given brain regions, such as amygdala, are monitored as central effects of OT in response to intranasal administration of OT, being detected by brain imaging devices. A part of such studies is listed in Table 2. However, none of reports deal with exact molecules involved in the OT transport, except for RAGE. Of course, it has been in part demonstrated and discussed the direct pathway to the brain from nasal cavity (the nose-brain route), intra-nerve transport, and the corpus callosum barrier at which the BBB is weaker. Anyhow, in such cases little or no molecular mechanisms have been reported.

**TABLE 2:** Identified transport molecules and routes of oxytocin to the brain after peripheral administration.

Method	Route	Crossing	Molecule	Brain regions	Spices	References
**(A) Animal studies**						
i.p.	Blood	BBB	–	CSF	Mouse	Neumann et al., 2013
i.p.	Blood	BBB	RAGE	CSF	Mouse	Yamamoto et al., 2019
s.c.	Blood	BBB	RAGE	CSF	Mouse	Yamamoto et al., 2019
i.v.	Blood	BBB	RAGE	PVN	Mouse	Yamamoto et al., 2019
i.n.	Blood*	BBB	RAGE	Amygdala	Mouse	Yamamoto et al., 2019
i.n.	Blood*	BBB	RAGE	mPFC	Mouse	Munesue et al., 2021
i.n.	–	–	–	Amygdala	Mouse	Smith et al., 2019
s.c.	Blood	–	–	Hippocampus	Mouse	Bertoni et al., 2021
i.n.	–	–		Olfactory bulb	Rat	Ferris et al., 2015
i.n.	–	–	–	CSF	Pig	Mens et al., 1983
i.n.	Olfactory N	–		Hypothalamus	Macaque	Lee et al., 2020
i.n.	Trigeminal N	–		Hypothalamus	Macaque	Lee et al., 2020
i.n.	–			CSF	Monkey	Modi et al., 2014
**(B) Human study**						
Oral	Blood	–	–	Putamen	Male	Kou et al., 2021
i.n.	Blood*	–	–	Amygdala	Male	Martins et al., 2020
i.n.	–	–	–	Amygdala	Male	Spengler et al., 2017
i.n.	–	–	–	Amygdala	Female	Lieberz et al., 2020
i.n.	–	–	–	Amygdala	Female	Geng et al., 2018
i.n.	Nose-to brain	–		Amygdala	Male	Quintana et al., 2017
i.n.	–	–	–	Amygdala	Male	Kirsch et al., 2005
i.n.	–	–	–	Striatum	M/F	Kirsch et al., 2005
I,n,	–	–	–	Temporal lobe	M/F	Frijling et al., 2016
i.n.	–	–	–	CSF	Male	Striepens et al., 2013
i.n.	–	–	–	Mirror neuron	Male	Ono et al., 2021

Blood indicates that OT is taken up into blood circulation. Blood * indicates that OT is mainly included in blood circulation after i.n. administration. However, other routes cannot be excluded. Nose-to-brain indicates the authors’ speculation that such routes exist. Blood routes are also possible but not specified or identified in each report. i.p., intraperitoneal; s.c., subcutaneous; i.v., intravenous; i.n., intranasal; BBB, blood-brain barrier; RAGE, receptors for advanced glycation end-products; CSF, cerebrospinal fluid; PVN, periventricular nucleus; mPFC, medial prefrontal cortex; N, nerve; and M/F, male and female.

## Functional Aspects of the Oxytocin Transport or Increases in the Brain

### Maintenance of the Brain Supply of Oxytocin in Postpartum Dams Is Critical

Gerasimenko et al. (2021) reported that maternal behavior, such as pup retrieval, was impaired in RAGE KO mice, as after additional stress, impairment of pup care was observed in mother mice. This neglect leads to newborn death within 1–2 days. RAGE seems to play a critical role during the postpartum period and is important in the manifestation of normal parental behavior in dams. Therefore, it can be proposed that RAGE-dependent OT signaling can dampen the effect of additional exogenous stress on endogenous stress during pregnancy, delivery, and lactation (Supplementary Figure 3). From these results in mice (Gerasimenko et al., 2021), in the early postpartum period in human females, a potential role for RAGE in transporting OT to the brain is crucial in the jumble of emotions during the puerperium and postpartum periods as suggested (Monks and Palanisamy, 2021). Thus, in the future, it will be necessary to examine whether RAGE impairment is more closely related to postpartum depression and child neglect, which is seen in 10 and 20%, respectively, of new human mothers.

### Nicotinamide Riboside

Finally, we discuss our recent results on a precursor of NAD (Chini et al., 2020, 2021). Similar to nicotinic acid and nicotinamide, nicotinamide riboside is a natural product found in milk. It is incorporated into the intracellular NAD pool. Nicotinamide riboside could be used as a general supplement, potentially for people who have adverse reactions to nicotinamide or nicotinamide riboside (Trammell et al., 2016). In brain tissue, NAD synthetase activity is dramatically low, making nicotinic acid an unsuitable supplement, as in the case of mice (Mori et al., 2014). However, nicotinamide riboside has already been used as a supplement or therapeutic agent to elevate or maintain cellular NAD content in mice (Fletcher et al., 2017).

Recently, it has been shown that NAD is consumed more by an increase in CD38 in aged male mice and, thus, proposed that inhibition of CD38 or an increase in NAD may lead to a longer life span (Tarragó et al., 2018; Gerasimenko et al., 2020a,b). NAD metabolism also catalyzes the formation of cADPR (Lee and Zhao, 2019). It then participates in OT release in the hypothalamus (Jin et al., 2007). Although NR supplementation did not change CD38 expression (Gerasimenko et al., 2020b), *in vitro* studies have shown that NAD applied to the mouse hypothalamus leads to OT release (Gerasimenko et al., 2020a).

It has been shown that daily administration of nicotinamide riboside ameliorated the social and behavioral impairments observed in male CD157 KO mice (Gerasimenko et al., 2020a). In contrast, an identical improvement in social behavior was not observed with saline gavage. Furthermore, nicotinamide riboside had essentially no effect on social behavior in wild-type mice. The increases in nicotinamide riboside treatment effects were only detected in CD157 KO mice. Therefore, these beneficial effects of nicotinamide riboside may be due to increased brain OT levels. No identical effect was observed with nicotinamide riboside in wild-type mice (Gerasimenko et al., 2020a).

## Conclusion

It has long been argued that intranasally applied OT in humans can be transmitted to the brain from the viewpoint of medical and psychological therapies. Our article series answers this, explaining that OT can be transported into the mouse brain *via* circulation. At the same time, we must recognize that concentrated nasal OT is an artificial situation. Humans have not encountered these conditions since the species’ origin several million years ago. Therefore, the recruitment of OT from the blood, in which the OT is present in the stream as a hormone that functions in remote places, to the brain is a more essential and natural condition for most mammals. This idea may be as important as the concept of OT release into the bloodstream of the pituitary. Harris and other researchers have reported this finding as a fundamental step in neuroendocrinology over a century ago (Leng et al., 2015). Moreover, we know now that mRAGE plays a role in OT transport in endothelial cells in mouse neurovascular units.

Biologically, it is possible to understand that the RAGE-dependent transport system may be a minimally safe system for supplying OT to the brain to maintain social behavior. Furthermore, the OT supply system from the periphery may provide information to the brain. However, this point needs to be further studied in the future.

Additional data have been reported concerning RAGE. First, OT concentrations in the CSF increase directly after absorption of OT from the intestine of mouse neonates upon oral administration of OT during only a few postnatal days. Second, during the 40 min after oral administration of the OT analog, LOT-1, RAGE is not converted into OT. Thus, it is not transported in the digestive system. Third, the three-dimensional image provides the precise location of RAGE in the mouse neurovascular endothelial cells, and Fourth, RAGE is located in human vascular endothelial cells. Through these novel data, in the current review, we evaluated the new concept that RAGE is mainly involved in the regulation of OT dynamics and the interface between the brain, blood, and intestine in the living body.

Finally, the RAGE-dependent OT transport hypothesis has been confirmed by measuring plasma OT concentrations by a simple extraction method called the PPT (acetonitrile protein precipitation) approach (Cherepanov et al., 2021). RAGE KO mice with less soluble forms of RAGE in the plasma displayed larger plasma OT concentration increases after intraperitoneal administration of OT. Furthermore, the changes were more extensive and lasted longer, proving that RAGE plays a role in OT dynamics in the blood.

## Author Contributions

HH, OH, YH, and YY wrote the manuscript. KFh, OL, MG, OH, YH, and HH draw figures. All authors performed experiments. All authors contributed to the article and approved the submitted version.

## Conflict of Interest

The authors declare that the research was conducted in the absence of any commercial or financial relationships that could be construed as a potential conflict of interest.

## Publisher’s Note

All claims expressed in this article are solely those of the authors and do not necessarily represent those of their affiliated organizations, or those of the publisher, the editors and the reviewers. Any product that may be evaluated in this article, or claim that may be made by its manufacturer, is not guaranteed or endorsed by the publisher.

## References

[B1] Avraham Y. Mankuta D. Lipsker L. Vorobiev L. Patael S. Hassid G. (2021). Beta-Carotene derivatives as novel therapy for the prevention and treatment of autistic symptoms. *Bioorg. Chem.* 115:105224. 10.1016/j.bioorg.2021.105224 34392174

[B2] Baumgartner T. Heinrichs M. Vonlanthen A. Fischbacher U. Fehr E. (2008). Oxytocin shapes the neural circuitry of trust and trust adaptation in humans. *Neuron* 58 639–650. 10.1016/j.neuron.2008.04.009 18498743

[B3] Beju D. Meek W. D. Kramer J. C. (2004). The ultrastructure of the nasal polyps in patients with and without cystic fibrosis. *J. Submicrosc. Cytol. Pathol.* 36 155–165. 15554502

[B4] Bertoni A. Schaller F. Tyzio R. Gaillard S. Santini F. Xolin M. (2021). Oxytocin administration in neonates shapes hippocampal circuitry and restores social behavior in a mouse model of autism. *Mol. Psychiatry* 26 7582–7595. 10.1038/s41380-021-01227-6 34290367 PMC8872977

[B5] Borowiak K. von Kriegstein K. (2020). Intranasal oxytocin modulates brain responses to voice-identity recognition in typically developing individuals, but not in ASD. *Transl. Psychiatry* 10:221. 10.1038/s41398-020-00903-5 32636360 PMC7341857

[B6] Braidy N. Berg J. Clement J. Khorshidi F. Poljak A. Jayasena T. (2019). Role of Nicotinamide Adenine Dinucleotide and Related Precursors as Therapeutic Targets for Age-Related Degenerative Diseases: Rationale. Biochemistry, Pharmacokinetics, and Outcomes. *Antioxid. Redox Signal.* 30 251–294. 10.1089/ars.2017.7269 29634344 PMC6277084

[B7] Bridges R. S. (2015). Neuroendocrine regulation of maternal behavior. *Front. Neuroendocrinol.* 36 178–196. 10.1016/j.yfrne.2014.11.007 25500107 PMC4342279

[B8] Camilleri M. (2019). Leaky gut: mechanisms, measurement and clinical implications in humans. *Gut* 68 1516–1526. 10.1136/gutjnl-2019-318427 31076401 PMC6790068

[B9] Carter C. S. (2022). Oxytocin and love: myths, metaphors and mysteries. *Comprehen. Psychoneuroendocrinol.* 9:100107. 10.1016/j.cpnec.2021.100107 PMC9216351 35755926

[B10] Carter C. S. Kenkel W. M. MacLean E. L. Wilson S. R. Perkeybile A. M. Yee J. R. (2020). Is Oxytocin “Nature’s Medicine”? *Pharmacol. Rev.* 72 829–861. 10.1124/pr.120.019398 32912963 PMC7495339

[B11] Chastant S. Mila H. (2019). Passive immune transfer in puppies. *Anim. Reprod. Sci.* 207 162–170. 10.1016/j.anireprosci.2019.06.012 31255495 PMC7125514

[B12] Cherepanov S. M. Akther S. Nishimura T. Shabalova A. A. Mizuno A. Ichinose W. (2017a). Effects of Three Lipidated Oxytocin Analogs on Behavioral Deficits in CD38 Knockout Mice. *Brain Sci.* 7:E132. 10.3390/brainsci7100132 29035307 PMC5664059

[B13] Cherepanov S. M. Yokoyama S. Mizuno A. Ichinose W. Lopatina O. Shabalova A. A. (2017b). Structure-specific effects of lipidated oxytocin analogs on intracellular calcium levels, parental behavior, and oxytocin concentrations in the plasma and cerebrospinal fluid in mice. *Pharmacol. Res. Perspect.* 5:e00290. 10.1002/prp2.290 28596839 PMC5461640

[B14] Cherepanov S. M. Gerasimenko M. Yuhi T. Shabalova A. Zhu H. Yokoyama S. (2021). An improved sample extraction method reveals that plasma receptor for advanced glycation end-products (RAGE) modulates circulating free oxytocin in mice. *Peptides* 146:170649. 10.1016/j.peptides.2021.170649 34543678

[B15] Cherepanov S. M. Miura R. Shabalova A. A. Ichinose W. Yokoyama S. Fukuda H. (2019). Synthesis of oxytocin derivatives lipidated via a carbonate or carbamate linkage as a long-acting therapeutic agent for social impairment-like behaviors. *Bioorg. Med. Chem.* 27 3358–3363. 10.1016/j.bmc.2019.06.018 31229420

[B16] Chini C. C. S. Peclat T. R. Warner G. M. Kashyap S. Espindola-Netto J. M. de Oliveira G. C. (2020). CD38 ecto-enzyme in immune cells is induced during aging and regulates NAD+ and NMN levels. *Nat. Metab.* 2 1284–1304. 10.1038/s42255-020-00298-z 33199925 PMC8752031

[B17] Chini C. C. S. Zeidler J. D. Kashyap S. Warner G. Chini E. N. (2021). Evolving concepts in NAD+ metabolism. *Cell Metab.* 33 1076–1087. 10.1016/j.cmet.2021.04.003 33930322 PMC8172449

[B18] Chong A. Malavasi F. Israel S. Khor C. C. Yap V. B. Monakhov M. (2017). ADP ribosyl-cyclases (CD38/CD157), social skills and friendship. *Psychoneuroendocrinology* 78 185–192. 10.1016/j.psyneuen.2017.01.011 28212520

[B19] Davis T. P. Abbruscato T. J. Egleton R. D. (2015). Peptides at the blood brain barrier: knowing me knowing you. *Peptides* 72 50–56. 10.1016/j.peptides.2015.04.020 25937599 PMC4627938

[B20] Ear P. H. Chadda A. Gumusoglu S. B. Schmidt M. S. Vogeler S. Malicoat J. (2019). Maternal Nicotinamide Riboside Enhances Postpartum Weight Loss, Juvenile Offspring Development, and Neurogenesis of Adult Offspring. *Cell Rep.* 22 969–983.e4. 10.1016/j.celrep.2019.01.007 30673618

[B21] Egleton R. D. Davis T. P. (2005). Development of neuropeptide drugs that cross the blood-brain barrier. *NeuroRx* 2 44–53. 10.1602/neurorx.2.1.44 15717056 PMC539319

[B22] Eskandari S. Varamini P. Toth I. (2013). Formulation, characterization and permeability study of nano particles of lipo-endomorphin-1 for oral delivery. *J. Liposome Res.* 23 311–317. 10.3109/08982104.2013.805339 23931529

[B23] Feldman R. (2017). The Neurobiology of Human Attachments. *Trends Cogn. Sci.* 21 80–99. 10.1016/j.tics.2016.11.007 28041836

[B24] Ferris C. F. Yee J. R. Kenkel W. M. Dumais K. M. Moore K. Veenema A. H. (2015). Distinct BOLD Activation Profiles Following Central and Peripheral Oxytocin Administration in Awake Rats. *Front. Behav. Neurosci.* 9:245. 10.3389/fnbeh.2015.00245 26441574 PMC4585275

[B25] Fletcher R. S. Ratajczak J. Doig C. L. Oakey L. A. Callingham R. Da Silva Xavier G. (2017). Nicotinamide riboside kinases display redundancy in mediating nicotinamide mononucleotide and nicotinamide riboside metabolism in skeletal muscle cells. *Mol. Metab.* 6 819–832. 10.1016/j.molmet.2017.05.011 28752046 PMC5518663

[B26] Frijling J. L. van Zuiden M. Koch S. B. J. Nawijn L. Veltman D. J. Olff M. (2016). Intranasal Oxytocin Affects Amygdala Functional Connectivity after Trauma Script-Driven Imagery in Distressed Recently Trauma-Exposed Individuals. *Neuropsychopharmacology* 41 1286–1296. 10.1038/npp.2015.278 26346640 PMC4793112

[B27] Froemke R. C. Young L. J. (2021). Oxytocin, Neural Plasticity, and Social Behavior. *Annu. Rev. Neurosci.* 44 359–381. 10.1146/annurev-neuro-102320-102847 33823654 PMC8604207

[B28] Geng Y. Zhao W. Zhou F. Ma X. Yao S. Becker B. (2018). Oxytocin Facilitates Empathic- and Self-embarrassment Ratings by Attenuating Amygdala and Anterior Insula Responses. *Front. Endocrinol.* 9:572. 10.3389/fendo.2018.00572 30356869 PMC6190868

[B29] Gerasimenko M. Cherepanov S. M. Furuhara K. Lopatina O. Salmina A. B. Shabalova A. A. (2020a). Nicotinamide riboside supplementation corrects deficits in oxytocin, sociability and anxiety of CD157 mutants in a mouse model of autism spectrum disorder. *Sci. Rep.* 10:10035. 10.1038/s41598-019-57236-7 32572044 PMC7308284

[B30] Gerasimenko M. Lopatina O. Shabalova A. A. Cherepanov S. M. Salmina A. B. Yokoyama S. (2020b). Distinct physical condition and social behavior phenotypes of CD157 and CD38 knockout mice during aging. *PLoS One* 15:e0244022. 10.1371/journal.pone.0244022 33326496 PMC7743928

[B31] Gerasimenko M. Lopatina O. Munesue S. Harashima A. Yokoyama S. Yamamoto Y. (2021). Receptor for advanced glycation end-products (RAGE) plays a critical role in retrieval behavior of mother mice at early postpartum. *Physiol. Behav.* 235:113395. 10.1016/j.physbeh.2021.113395 33757778

[B32] Grinevich V. Neumann I. D. (2021). Brain oxytocin: how puzzle stones from animal studies translate into psychiatry. *Mol. Psychiatry* 26 265–279. 10.1038/s41380-020-0802-9 32514104 PMC7278240

[B33] Guastella A. J. Cooper M. N. White C. R. H. White M. K. Pennell C. E. Whitehouse A. J. O. (2018). Does perinatal exposure to exogenous oxytocin influence child behavioural problems and autistic-like behaviours to 20 years of age? *J. Child Psychol. Psychiatry* 59 1323–1332. 10.1111/jcpp.12924 29701247

[B34] Harashima A. Yamamoto Y. Cheng C. Tsuneyama K. Myint K. M. Takeuchi A. (2006). Identification of mouse orthologue of endogenous secretory receptor for advanced glycation end-products: structure, function and expression. *Biochem. J.* 396 109–115. 10.1042/BJ20051573 16503878 PMC1450004

[B35] Higashida H. (2016). Somato-axodendritic release of oxytocin into the brain due to calcium amplification is essential for social memory. *J. Physiol. Sci. JPS* 66 275–282. 10.1007/s12576-015-0425-0 26586001 PMC4893072

[B36] Higashida H. Liang M. Yoshihara T. Akther S. Fakhrul A. Stanislav C. (2017b). An immunohistochemical, enzymatic, and behavioral study of CD157/BST-1 as a neuroregulator. *BMC Neurosci.* 18:35. 10.1186/s12868-017-0350-7 28340569 PMC5366154

[B37] Higashida H. Furuhara K. Yamauchi A.-M. Deguchi K. Harashima A. Munesue S. (2017a). Intestinal transepithelial permeability of oxytocin into the blood is dependent on the receptor for advanced glycation end products in mice. *Sci. Rep.* 7:7883. 10.1038/s41598-017-07949-4 28801574 PMC5554167

[B38] Higashida H. Hashii M. Tanaka Y. Matsukawa S. Higuchi Y. Gabata R. (2019a). CD38, CD157, and RAGE as Molecular Determinants for Social Behavior. *Cells* 9:E62. 10.3390/cells9010062 31881755 PMC7016687

[B39] Higashida H. Munesue T. Kosaka H. Yamasue H. Yokoyama S. Kikuchi M. (2019b). Social Interaction Improved by Oxytocin in the Subclass of Autism with Comorbid Intellectual Disabilities. *Diseases* 7:E24. 10.3390/diseases7010024 30813294 PMC6473850

[B40] Higashida H. Yokoyama S. Munesue T. Kikuchi M. Minabe Y. Lopatina O. (2011). CD38 gene knockout juvenile mice: a model of oxytocin signal defects in autism. *Biol. Pharm. Bull.* 34 1369–1372. 10.1248/bpb.34.1369 21881219

[B41] Higashida H. Yuhi T. Akther S. Amina S. Zhong J. Liang M. (2018). Oxytocin release via activation of TRPM2 and CD38 in the hypothalamus during hyperthermia in mice: implication for autism spectrum disorder. *Neurochem. Int.* 119 42–48. 10.1016/j.neuint.2017.07.009 28736241

[B42] Hu Z. Fang W. Liu Y. Liang H. Chen W. Wang H. (2021). Acute glucose fluctuation promotes RAGE expression via reactive oxygen species-mediated NF-κB activation in rat podocytes. *Mol. Med. Rep.* 23:330. 10.3892/mmr.2021.11969 33760170 PMC7974412

[B43] Huang Y. Huang X. Ebstein R. P. Yu R. (2021). Intranasal oxytocin in the treatment of autism spectrum disorders: a multilevel meta-analysis. *Neurosci. Biobehav. Rev.* 122 18–27. 10.1016/j.neubiorev.2020.12.028 33400920

[B44] Huang Y. Kendrick K. M. Zheng H. Yu R. (2015). Oxytocin enhances implicit social conformity to both in-group and out-group opinions. *Psychoneuroendocrinology* 60 114–119. 10.1016/j.psyneuen.2015.06.003 26143536

[B45] Ichinose W. Cherepanov S. M. Shabalova A. A. Yokoyama S. Yuhi T. Yamaguchi H. (2019). Development of a Highly Potent Analogue and a Long-Acting Analogue of Oxytocin for the Treatment of Social Impairment-Like Behaviors. *J. Med. Chem.* 62 3297–3310. 10.1021/acs.jmedchem.8b01691 30896946

[B46] Jin D. Liu H.-X. Hirai H. Torashima T. Nagai T. Lopatina O. (2007). CD38 is critical for social behaviour by regulating oxytocin secretion. *Nature* 446 41–45. 10.1038/nature05526 17287729

[B47] Jurek B. Neumann I. D. (2018). The Oxytocin Receptor: From Intracellular Signaling to Behavior. *Physiol. Rev.* 98 1805–1908. 10.1152/physrev.00031.2017 29897293

[B48] Kasai S. Yoshihara T. Lopatina O. Ishihara K. Higashida H. (2017). Selegiline Ameliorates Depression-Like Behavior in Mice Lacking the CD157/BST1 Gene, a Risk Factor for Parkinson’s Disease. *Front. Behav. Neurosci.* 11:75. 10.3389/fnbeh.2017.00075 28515684 PMC5413561

[B49] Kirsch P. Esslinger C. Chen Q. Mier D. Lis S. Siddhanti S. (2005). Oxytocin modulates neural circuitry for social cognition and fear in humans. *J. Neurosci.* 25 11489–11493. 10.1523/JNEUROSCI.3984-05.2005 16339042 PMC6725903

[B50] Kou J. Lan C. Zhang Y. Wang Q. Zhou F. Zhao Z. (2021). In the nose or on the tongue? Contrasting motivational effects of oral and intranasal oxytocin on arousal and reward during social processing. *Transl. Psychiatry* 11:94. 10.1038/s41398-021-01241-w 33542175 PMC7862637

[B51] Kouidrat Y. Amad A. Arai M. Miyashita M. Lalau J. D. Loas G. (2015). *J. Psychiatr. Res.* 66-67 112–117. 10.1016/j.jpsychires.2015.04.023 26001588

[B52] Krol K. M. Grossmann T. (2018). Psychological effects of breastfeeding on children and mothers. *Bundesgesundheitsblatt Gesundheitsforschung Gesundheitsschutz* 61 977–985. 10.1007/s00103-018-2769-0 29934681 PMC6096620

[B53] Kurokawa H. Kinari Y. Okudaira H. Tsubouchi K. Sai Y. Kikuchi M. (2020). Competitiveness and individual characteristics: a double-blind placebo-controlled study using oxytocin. *Sci. Rep.* 10:11526. 10.1038/s41598-020-68445-w 32661293 PMC7359354

[B54] Kurokawa H. Kinari Y. Okudaira H. Tsubouchi K. Sai Y. Kikuchi M. (2021). Oxytocin-Trust Link in Oxytocin-Sensitive Participants and Those Without Autistic Traits. *Front. Neurosci.* 15:659737. 10.3389/fnins.2021.659737 34113232 PMC8186783

[B55] Lakkireddy H. R. Urmann M. Besenius M. Werner U. Haack T. Brun P. (2016). Oral delivery of diabetes peptides - Comparing standard formulations incorporating functional excipients and nanotechnologies in the translational context. *Adv. Drug Deliv. Rev.* 106 196–222. 10.1016/j.addr.2016.02.011 26964477

[B56] Lee H. C. Zhao Y. J. (2019). Resolving the topological enigma in Ca^2+^ signaling by cyclic ADP-ribose and NAADP. *J. Biol. Chem.* 294 19831–19843. 10.1074/jbc.REV119.009635 31672920 PMC6937575

[B57] Lee M. R. Jayant R. D. (2019). Penetration of the blood-brain barrier by peripheral neuropeptides: new approaches to enhancing transport and endogenous expression. *Cell Tissue Res.* 375 287–293. 10.1007/s00441-018-2959-y 30535799 PMC6467522

[B58] Lee M. R. Shnitko T. A. Blue S. W. Kaucher A. V. Winchell A. J. Erikson D. W. (2020). Labeled oxytocin administered via the intranasal route reaches the brain in rhesus macaques. *Nat. Commun.* 11:2783. 10.1038/s41467-020-15942-1 32494001 PMC7270110

[B59] Leerach N. Harashima A. Munesue S. Kimura K. Oshima Y. Goto H. (2021). Glycation reaction and the role of the receptor for advanced glycation end-products in immunity and social behavior. *Glycoconj. J.* 38 303–310. 10.1007/s10719-020-09956-6 33108607

[B60] Leng G. Pineda R. Sabatier N. Ludwig M. (2015). 60 YEARS OF NEUROENDOCRINOLOGY: The posterior pituitary, from Geoffrey Harris to our present understanding. *J. Endocrinol.* 226 T173–T185. 10.1530/JOE-15-0087 25901040

[B61] Lieberz J. Scheele D. Spengler F. B. Matheisen T. Schneider L. Stoffel-Wagner B. (2020). Kinetics of oxytocin effects on amygdala and striatal reactivity vary between women and men. *Neuropsychopharmacology* 45 1134–1140. 10.1038/s41386-019-0582-6 31785587 PMC7235226

[B62] Lopatina O. Yoshihara T. Nishimura T. Zhong J. Akther S. Fakhrul A. A. (2014). Anxiety- and depression-like behavior in mice lacking the CD157/BST1 gene, a risk factor for Parkinson’s disease. *Front. Behav. Neurosci.* 8:133. 10.3389/fnbeh.2014.00133 24795584 PMC4001052

[B63] Mackic J. B. Stins M. McComb J. G. Calero M. Ghiso J. Kim K. S. (1998). Human blood-brain barrier receptors for Alzheimer’s amyloid-beta 1- 40. Asymmetrical binding, endocytosis, and transcytosis at the apical side of brain microvascular endothelial cell monolayer. *J. Clin. Invest.* 102 734–743. 10.1172/JCI2029 9710442 PMC508936

[B64] Maejima Y. Horita S. Otsuka A. Hidema S. Nishimori K. Shimomura K. (2020). Oral oxytocin delivery with proton pump inhibitor pretreatment decreases food intake. *Peptides* 128:170312. 10.1016/j.peptides.2020.170312 32298773

[B65] Manigrasso M. B. Friedman R. A. Ramasamy R. D’Agati V. Schmidt A. M. (2018). Deletion of the formin Diaph1 protects from structural and functional abnormalities in the murine diabetic kidney. *Am. J. Physiol. Renal Physiol.* 315 F1601–F1612. 10.1152/ajprenal.00075.2018 30132346 PMC6336994

[B66] Martins D. A. Mazibuko N. Zelaya F. Vasilakopoulou S. Loveridge J. Oates A. (2020). Effects of route of administration on oxytocin-induced changes in regional cerebral blood flow in humans. *Nat. Commun.* 11:1160. 10.1038/s41467-020-14845-5 32127545 PMC7054359

[B67] Mens W. B. Witter A. van Wimersma Greidanus T. B. (1983). Penetration of neurohypophyseal hormones from plasma into cerebrospinal fluid (CSF): half-times of disappearance of these neuropeptides from CSF. *Brain Res.* 262 143–149. 10.1016/0006-8993(83)90478-x 6831225

[B68] Mizuno A. Cherepanov S. M. Kikuchi Y. Fakhrul A. A. Akther S. Deguchi K. (2015). Lipo-oxytocin-1, a Novel Oxytocin Analog Conjugated with Two Palmitoyl Groups, Has Long-Lasting Effects on Anxiety-Related Behavior and Social Avoidance in CD157 Knockout Mice. *Brain Sci.* 5 3–13. 10.3390/brainsci5010003 25612002 PMC4390788

[B69] Modi M. E. Connor-Stroud F. Landgraf R. Young L. J. Parr L. A. (2014). Aerosolized oxytocin increases cerebrospinal fluid oxytocin in rhesus macaques. *Psychoneuroendocrinology* 45 49–57. 10.1016/j.psyneuen.2014.02.011 24845176 PMC4120060

[B70] Monks D. T. Palanisamy A. (2021). Oxytocin: at birth and beyond. A systematic review of the long-term effects of peripartum oxytocin. *Anaesthesia* 76 1526–1537. 10.1111/anae.15553 34389972

[B71] Mori M. Ishikawa G. Takeshita T. Goto T. Robinson J. M. Takizawa T. (2006). Ultrahigh-resolution immunofluorescence microscopy using ultrathin cryosections: subcellular distribution of caveolin-1alpha and CD31 in human placental endothelial cells. *J. Electron Microsc.* 55, 107–112. 10.1093/jmicro/dfl011 16670105

[B72] Mori V. Amici A. Mazzola F. Di Stefano M. Conforti L. Magni G. (2014). Metabolic profiling of alternative NAD biosynthetic routes in mouse tissues. *PLoS One* 9:e113939. 10.1371/journal.pone.0113939 25423279 PMC4244216

[B73] Munesue S.-I. Liang M. Harashima A. Zhong J. Furuhara K. Boitsova E. B. (2021). Transport of oxytocin to the brain after peripheral administration by membrane-bound or soluble forms of receptors for advanced glycation end-products. *J. Neuroendocrinol.* 33:e12963. 10.1111/jne.12963 33733541

[B74] Munesue T. Nakamura H. Kikuchi M. Miura Y. Takeuchi N. Anme T. (2016). Oxytocin for Male Subjects with Autism Spectrum Disorder and Comorbid Intellectual Disabilities: A Randomized Pilot Study. *Front. Psychiatry* 7:2. 10.3389/fpsyt.2016.00002 26834651 PMC4720778

[B75] Munesue T. Yokoyama S. Nakamura K. Anitha A. Yamada K. Hayashi K. (2010). Two genetic variants of CD38 in subjects with autism spectrum disorder and controls. *Neurosci. Res.* 67 181–191. 10.1016/j.neures.2010.03.004 20435366

[B76] Muranishi Y. Parry L. Averous J. Terrisse A. Maurin A.-C. Chaveroux C. (2016). Method for collecting mouse milk without exogenous oxytocin stimulation. *BioTechniques* 60 47–49. 10.2144/000114373 26757812

[B77] Neumann I. D. Maloumby R. Beiderbeck D. I. Lukas M. Landgraf R. (2013). Increased brain and plasma oxytocin after nasal and peripheral administration in rats and mice. *Psychoneuroendocrinology* 38 1985–1993. 10.1016/j.psyneuen.2013.03.003 23579082

[B78] Nielsen S. D. Beverly R. L. Underwood M. A. Dallas D. C. (2020). Differences and Similarities in the Peptide Profile of Preterm and Term Mother’s Milk, and Preterm and Term Infant Gastric Samples. *Nutrients* 12:E2825. 10.3390/nu12092825 32942688 PMC7551100

[B79] Nikiforov A. Kulikova V. Ziegler M. (2015). The human NAD metabolome: functions, metabolism and compartmentalization. *Crit. Rev. Biochem. Mol. Biol.* 50 284–297. 10.3109/10409238.2015.1028612 25837229 PMC4673589

[B80] Noel G. In J. G. Lemme-Dumit J. M. DeVine L. R. Cole R. N. Guerrerio A. L. (2021). Human Breast Milk Enhances Intestinal Mucosal Barrier Function and Innate Immunity in a Healthy Pediatric Human Enteroid Model. *Front. Cell Dev. Biol.* 9:685171. 10.3389/fcell.2021.685171 34327199 PMC8313895

[B81] Noguchi Y. Maeda A. Wang H.-T. Takakura C. Lo P.-C. Kodama T. (2020). Human CD31 on Swine Endothelial Cells Induces SHP-1 Phosphorylation in Macrophages. *Transplant. Proc.* 52 1913–1915. 10.1016/j.transproceed.2020.01.140 32402461

[B82] Numan M. Young L. J. (2016). Neural mechanisms of mother-infant bonding and pair bonding: similarities, differences, and broader implications. *Horm. Behav.* 77 98–112. 10.1016/j.yhbeh.2015.05.015 26062432 PMC4671834

[B83] Okamoto H. Takasawa S. (2021). Okamoto model for necrosis and its expansions, CD38-cyclic ADP-ribose signal system for intracellular Ca^2+^ mobilization and Reg (Regenerating gene protein)-Reg receptor system for cell regeneration. *Proc. Jpn. Acad. Ser. B Phys. Biol. Sci.* 97 423–461. 10.2183/pjab.97.022 34629354 PMC8553518

[B84] Okamoto Y. Ishitobi M. Wada Y. Kosaka H. (2016). The Potential of Nasal Oxytocin Administration for Remediation of Autism Spectrum Disorders. *CNS Neurol. Disord. Drug Targets* 15 564–577. 10.2174/1871527315666160413120845 27071789 PMC5080861

[B85] Ono Y. Hirosawa T. Hasegawa C. Ikeda T. Kudo K. Naito N. (2021). Influence of oxytocin administration on somatosensory evoked magnetic fields induced by median nerve stimulation during hand action observation in healthy male volunteers. *PLoS One* 16:e0249167. 10.1371/journal.pone.0249167 33788881 PMC8011787

[B86] Quintana D. S. Guastella A. J. (2020). An Allostatic Theory of Oxytocin. *Trends Cogn. Sci.* 24 515–528. 10.1016/j.tics.2020.03.008 32360118

[B87] Quintana D. S. Smerud K. T. Andreassen O. A. Djupesland P. G. (2018). Evidence for intranasal oxytocin delivery to the brain: recent advances and future perspectives. *Ther. Deliv.* 9 515–525. 10.4155/tde-2018-0002 29943688

[B88] Quintana D. S. Westlye L. T. Hope S. Nærland T. Elvsåshagen T. Dørum E. (2017). Dose-dependent social-cognitive effects of intranasal oxytocin delivered with novel Breath Powered device in adults with autism spectrum disorder: a randomized placebo-controlled double-blind crossover trial. *Transl. Psychiatry* 7:e1136. 10.1038/tp.2017.103 28534875 PMC5584522

[B89] Raman K. G. Sappington P. L. Yang R. Levy R. M. Prince J. M. Liu S. (2006). The role of RAGE in the pathogenesis of intestinal barrier dysfunction after hemorrhagic shock. *Am. J. Physiol. Gastrointest. Liver Physiol.* 291 G556–G565. 10.1152/ajpgi.00055.2006 16751175

[B90] Ramirez M. I. Pollack L. Millien G. Cao Y. X. Hinds A. Williams M. C. (2002). The alpha-isoform of caveolin-1 is a marker of vasculogenesis in early lung development. *J. Histochem. Cytochem.* 50 33–42. 10.1177/002215540205000104 11748292

[B91] Rault J.-L. (2016). Effects of positive and negative human contacts and intranasal oxytocin on cerebrospinal fluid oxytocin. *Psychoneuroendocrinology* 69 60–66. 10.1016/j.psyneuen.2016.03.015 27032064

[B92] Roca Rubio M. F. Eriksson U. Brummer R. J. König J. (2021). Sauna dehydration as a new physiological challenge model for intestinal barrier function. *Sci. Rep.* 11:15514. 10.1038/s41598-021-94814-0 34330970 PMC8324874

[B93] Russell J. A. (2018). Fifty Years of Advances in Neuroendocrinology. *Brain Neurosci. Adv.* 2:2398212818812014. 10.1177/2398212818812014 32166160 PMC7058251

[B94] Russell J. A. Leng G. Douglas A. J. (2003). The magnocellular oxytocin system, the fount of maternity: adaptations in pregnancy. *Front. Neuroendocrinol.* 24 27–61. 10.1016/s0091-3022(02)00104-8 12609499

[B95] Schmidt A. M. Stern D. M. (2001). Receptor for age (RAGE) is a gene within the major histocompatibility class III region: implications for host response mechanisms in homeostasis and chronic disease. *Front. Biosci. J. Virtual Libr.* 6 D1151–D1160. 10.2741/schmidt 11578972

[B96] Schmidt A. M. Yan S. D. Yan S. F. Stern D. M. (2000). The biology of the receptor for advanced glycation end products and its ligands. *Biochim. Biophys. Acta* 1498 99–111. 10.1016/s0167-4889(00)00087-2 11108954

[B97] Shimizu Y. Harashima A. Munesue S. Oishi M. Hattori T. Hori O. (2020). Neuroprotective Effects of Endogenous Secretory Receptor for Advanced Glycation End-products in Brain Ischemia. *Aging Dis.* 11 547–558. 10.14336/AD.2019.0715 32489701 PMC7220285

[B98] Smith A. S. Korgan A. C. Young W. S. (2019). Oxytocin delivered nasally or intraperitoneally reaches the brain and plasma of normal and oxytocin knockout mice. *Pharmacol. Res.* 146:104324. 10.1016/j.phrs.2019.104324 31238093 PMC6679720

[B99] Spengler F. B. Schultz J. Scheele D. Essel M. Maier W. Heinrichs M. (2017). Kinetics and Dose Dependency of Intranasal Oxytocin Effects on Amygdala Reactivity. *Biol. Psychiatry* 82 885–894. 10.1016/j.biopsych.2017.04.015 28629540

[B100] Stern D. M. Yan S. D. Yan S. F. Schmidt A. M. (2002). Receptor for advanced glycation endproducts (RAGE) and the complications of diabetes. *Ageing Res. Rev.* 1 1–15. 10.1016/s0047-6374(01)00366-9 12039445

[B101] Stern D. Yan S. D. Yan S. F. Schmidt A. M. (2002). Receptor for advanced glycation endproducts: a multiligand receptor magnifying cell stress in diverse pathologic settings. *Adv. Drug Deliv. Rev.* 54 1615–1625. 10.1016/s0169-409x(02)00160-6 12453678

[B102] Stevenson A. J. Vanwalleghem G. Stewart T. A. Condon N. D. Lloyd-Lewis B. Marino N. (2020). Multiscale imaging of basal cell dynamics in the functionally mature mammary gland. *Proc. Natl. Acad. Sci. U. S. A.* 117 26822–26832. 10.1073/pnas.2016905117 33033227 PMC7604439

[B103] Strasser F. J. Feldmann M. Gross J. J. Müller A. T. M. Pfingstner H. Corti S. (2021). Pathogen dependent effects of high amounts of oxytocin on the bloodmilk barrier integrity during mastitis in dairy cows. *Schweiz. Arch. Tierheilkd.* 163 327–337. 10.17236/sat00302 33941509

[B104] Striepens N. Kendrick K. M. Hanking V. Landgraf R. Wüllner U. Maier W. (2013). Elevated cerebrospinal fluid and blood concentrations of oxytocin following its intranasal administration in humans. *Sci. Rep.* 3:3440. 10.1038/srep03440 24310737 PMC3853684

[B105] Takeda S. Kuwabara Y. Mizuno M. (1986). Concentrations and origin of oxytocin in breast milk. *Endocrinol. Jpn.* 33 821–826. 10.1507/endocrj1954.33.821 3582266

[B106] Tanaka A. Furubayashi T. Arai M. Inoue D. Kimura S. Kiriyama A. (2018). Delivery of Oxytocin to the Brain for the Treatment of Autism Spectrum Disorder by Nasal Application. *Mol. Pharm.* 15 1105–1111. 10.1021/acs.molpharmaceut.7b00991 29338251

[B107] Tarragó M. G. Chini C. C. S. Kanamori K. S. Warner G. M. Caride A. de Oliveira G. C. (2018). A Potent and Specific CD38 Inhibitor Ameliorates Age-Related Metabolic Dysfunction by Reversing Tissue NAD+ Decline. *Cell Metab.* 27 1081–1095.e10. 10.1016/j.cmet.2018.03.016 29719225 PMC5935140

[B108] Tolomeo S. Chiao B. Lei Z. Chew S. H. Ebstein R. P. (2020). A Novel Role of CD38 and Oxytocin as Tandem Molecular Moderators of Human Social Behavior. *Neurosci. Biobehav. Rev.* 115 251–272. 10.1016/j.neubiorev.2020.04.013 32360414

[B109] Trammell S. A. J. Schmidt M. S. Weidemann B. J. Redpath P. Jaksch F. Dellinger R. W. (2016). Nicotinamide riboside is uniquely and orally bioavailable in mice and humans. *Nat. Commun.* 7:12948. 10.1038/ncomms12948 27721479 PMC5062546

[B110] Veening J. G. Olivier B. (2013). Intranasal administration of oxytocin: behavioral and clinical effects, a review. *Neurosci. Biobehav. Rev.* 37 1445–1465. 10.1016/j.neubiorev.2013.04.012 23648680 PMC7112651

[B111] Wellnitz O. Bruckmaier R. M. (2021). Invited review: the role of the blood-milk barrier and its manipulation for the efficacy of the mammary immune response and milk production. *J. Dairy Sci.* 104 6376–6388. 10.3168/jds.2020-20029 33773785

[B112] Xu R. J. Wang T. (1996). Gastrointestinal absorption of insulinlike growth factor-I in neonatal pigs. *J. Pediatr. Gastroenterol. Nutr.* 23 430–437. 10.1097/00005176-199611000-00013 8956182

[B113] Yamamoto H. Watanabe T. Yamamoto Y. Yonekura H. Munesue S. Harashima A. (2007). RAGE in diabetic nephropathy. *Curr. Mol. Med.* 7 752–757. 10.2174/156652407783220769 18331233

[B114] Yamamoto Y. Higashida H. (2020). RAGE regulates oxytocin transport into the brain. *Commun. Biol.* 3:70. 10.1038/s42003-020-0799-2 32054984 PMC7018824

[B115] Yamamoto Y. Kato I. Doi T. Yonekura H. Ohashi S. Takeuchi M. (2001). Development and prevention of advanced diabetic nephropathy in RAGE-overexpressing mice. *J. Clin. Invest.* 108 261–268. 10.1172/JCI11771 11457879 PMC203021

[B116] Yamamoto Y. Liang M. Munesue S. Deguchi K. Harashima A. Furuhara K. (2019). Vascular RAGE transports oxytocin into the brain to elicit its maternal bonding behaviour in mice. *Commun. Biol.* 2:76. 10.1038/s42003-019-0325-6 30820471 PMC6389896

[B117] Yeomans D. C. Hanson L. R. Carson D. S. Tunstall B. J. Lee M. R. Tzabazis A. Z. (2021). Nasal oxytocin for the treatment of psychiatric disorders and pain: achieving meaningful brain concentrations. *Transl. Psychiatry* 11:388. 10.1038/s41398-021-01511-7 34247185 PMC8272715

[B118] Yonekura H. Yamamoto Y. Sakurai S. Petrova R. G. Abedin M. J. Li H. (2003). Novel splice variants of the receptor for advanced glycation end-products expressed in human vascular endothelial cells and pericytes, and their putative roles in diabetes-induced vascular injury. *Biochem. J.* 370 1097–1109. 10.1042/BJ20021371 12495433 PMC1223244

[B119] Yonekura H. Yamamoto Y. Sakurai S. Watanabe T. Yamamoto H. (2005). Roles of the receptor for advanced glycation endproducts in diabetes-induced vascular injury. *J. Pharmacol. Sci.* 97 305–311. 10.1254/jphs.cpj04005x 15750291

[B120] Zheng X. Kendrick K. M. (2021). Neural and Molecular Contributions to Pathological Jealousy and a Potential Therapeutic Role for Intranasal Oxytocin. *Front. Pharmacol.* 12:652473. 10.3389/fphar.2021.652473 33959017 PMC8094533

