# Supplementary material for: Oxytocin Dynamics in the Body and Brain Regulated by the Receptor for Advanced Glycation End-Products, CD38, CD157, and Nicotinamide Riboside
Source: Front Neurosci. 2022 Jul 7;16:858070. doi: 10.3389/fnins.2022.858070 (PMC9301327; doi:10.3389/fnins.2022.858070)
Supplement: Supplementary file 1 [file Presentation_1.PPTX]

## Slide 1
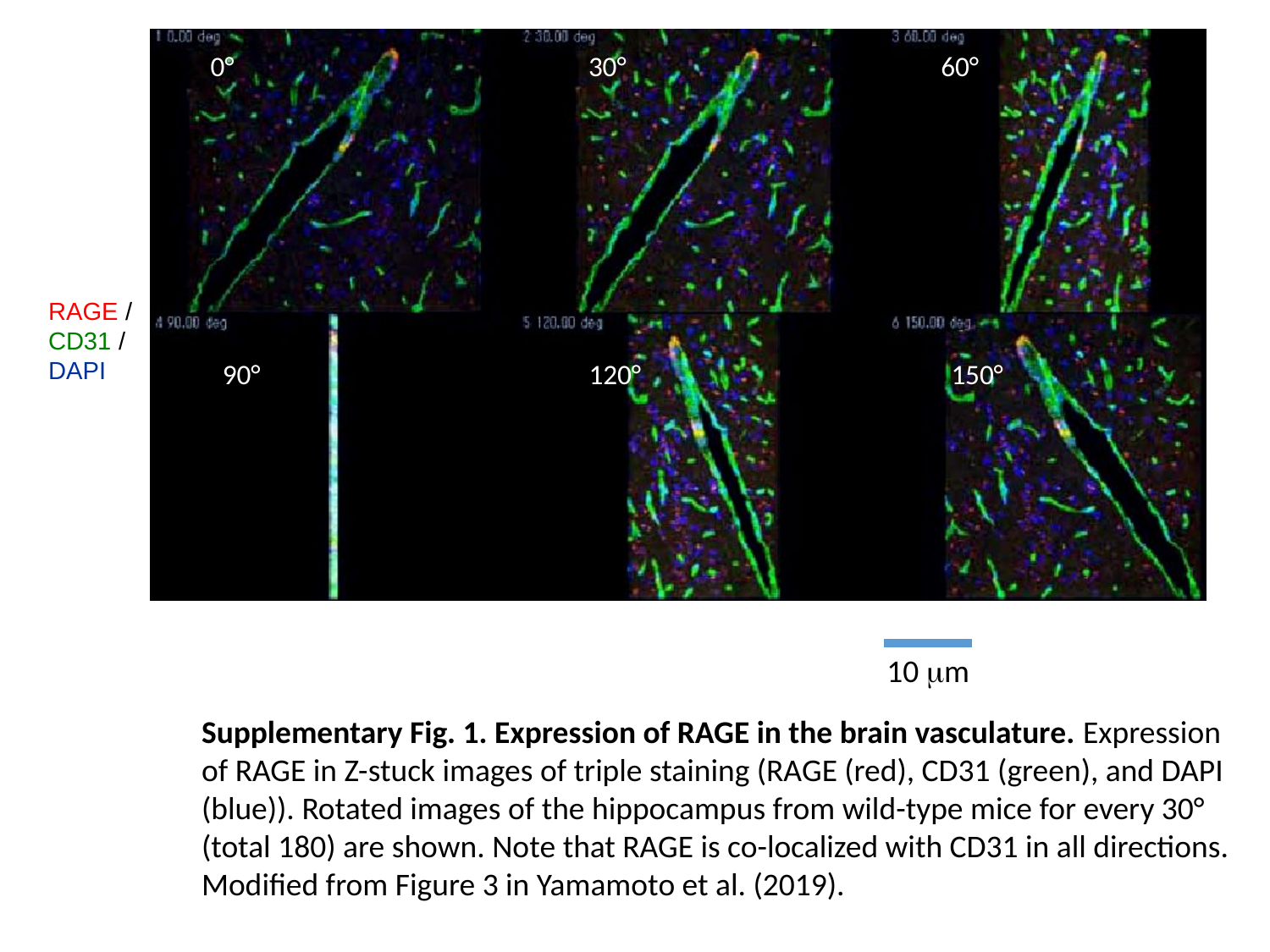

0°
30°
60°
RAGE /
CD31 /
DAPI
90°
120°
150°
10 mm
Supplementary Fig. 1. Expression of RAGE in the brain vasculature. Expression of RAGE in Z-stuck images of triple staining (RAGE (red), CD31 (green), and DAPI (blue)). Rotated images of the hippocampus from wild-type mice for every 30° (total 180) are shown. Note that RAGE is co-localized with CD31 in all directions.
Modified from Figure 3 in Yamamoto et al. (2019).
Rage+/+
Rage-/-
Extended Data Figure 2. Z-stuck analysis for RAGE expression and immunoelectron microscopical analysis for OT expression. a,b. Expression of Rage in Z-stuck images. Z-stuck images of triple staining (Rage, CD31 and DAPI) in Fig. 5 a-d were obtained, and rotated by every 30° (total 180°). Note that Rage is co-localized with CD31 in all directions. c,d. Immunogold localization of OT in the secretory vesicles in the posterior pituitary in both of Rage+/+ and Rage-/- mice.

## Slide 2
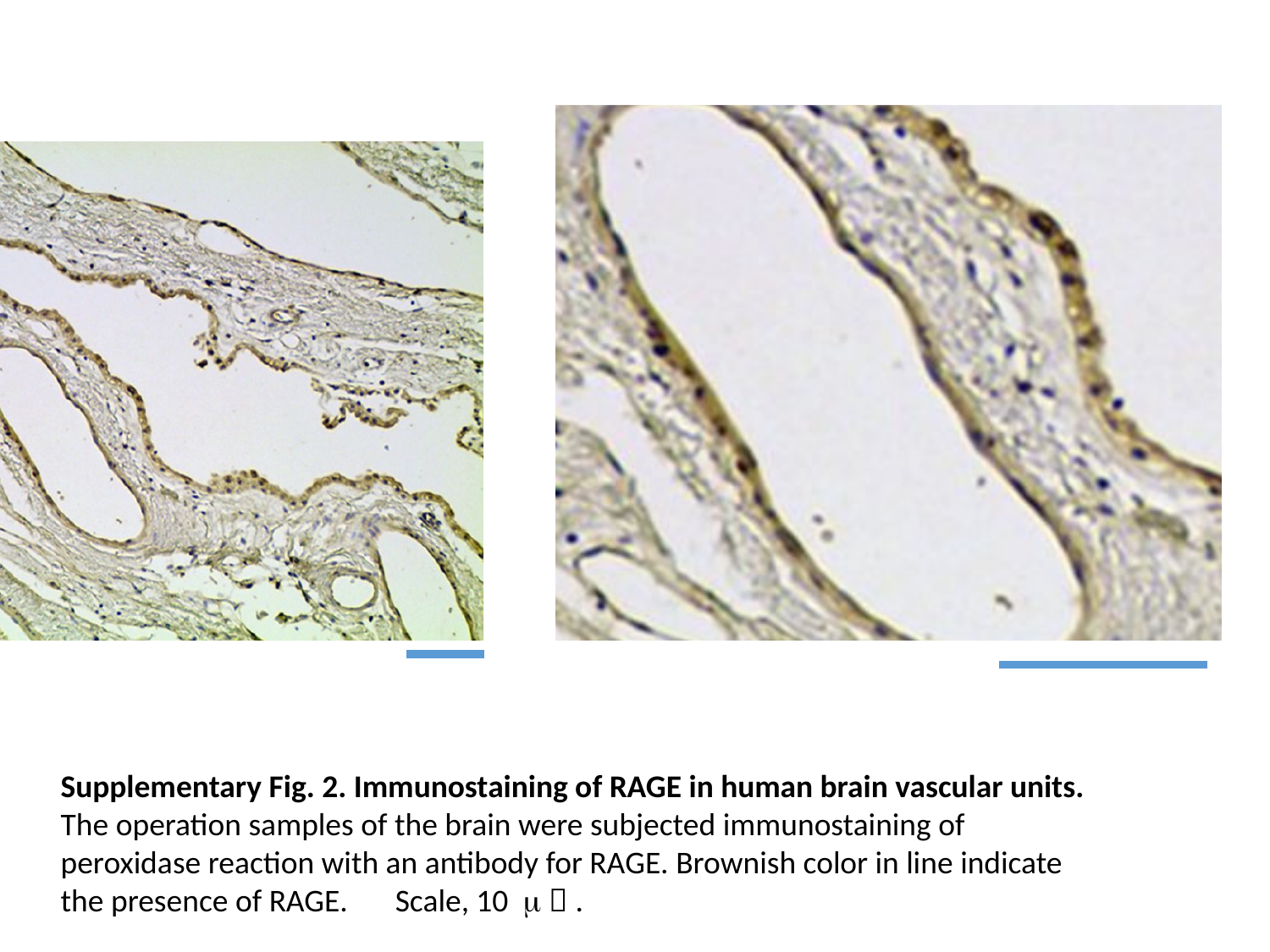

Extended Data Figure 7. Immunostaining for RAGE in human choroid plexus.
Supplementary Fig. 2. Immunostaining of RAGE in human brain vascular units. The operation samples of the brain were subjected immunostaining of peroxidase reaction with an antibody for RAGE. Brownish color in line indicate the presence of RAGE.　Scale, 10 mｍ.

## Slide 3
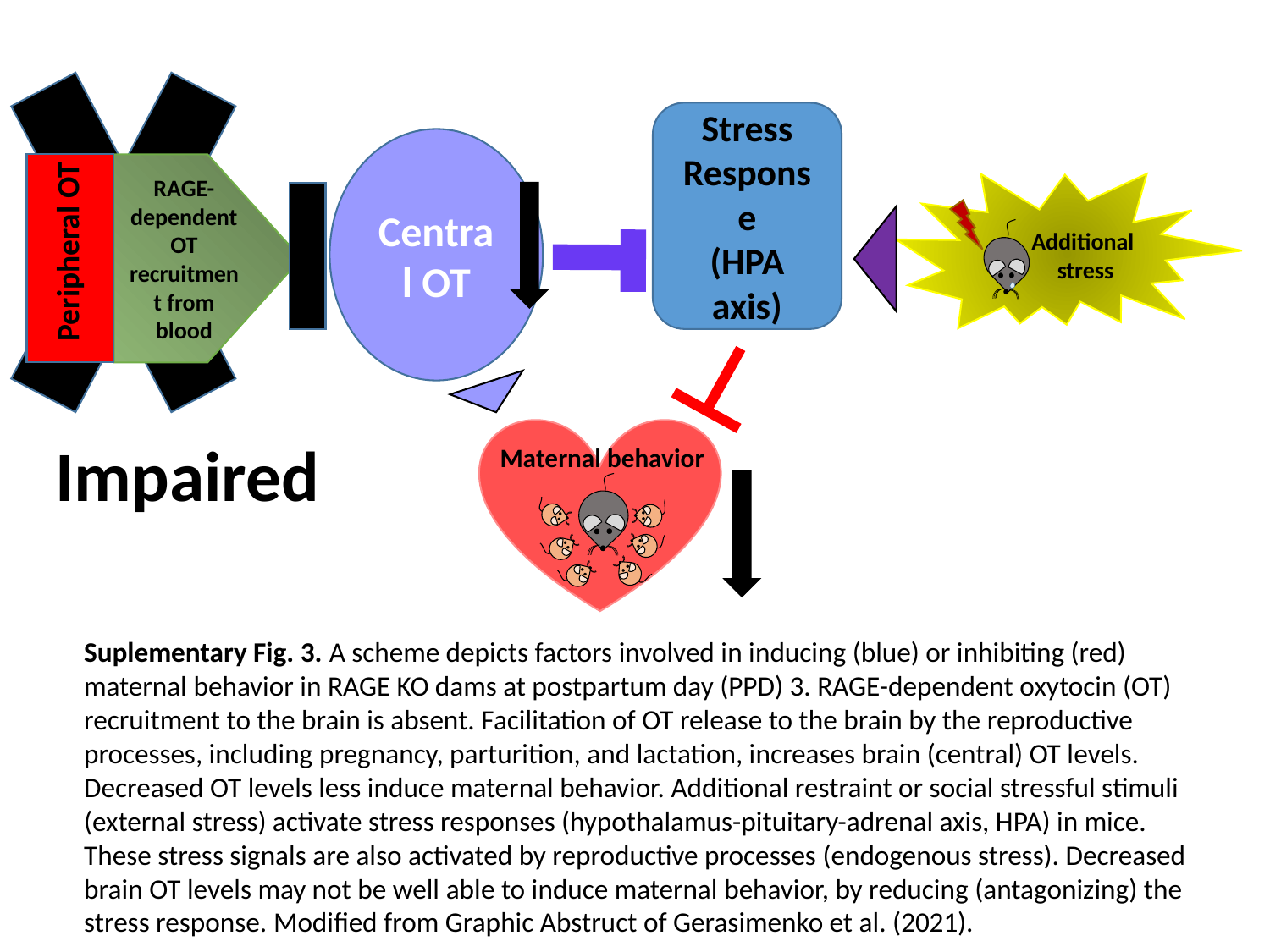

Stress Response
(HPA axis)
Central OT
RAGE-dependent OT recruitment from blood
Additional
 stress
Peripheral OT
Maternal behavior
Impaired
Suplementary Fig. 3. A scheme depicts factors involved in inducing (blue) or inhibiting (red) maternal behavior in RAGE KO dams at postpartum day (PPD) 3. RAGE-dependent oxytocin (OT) recruitment to the brain is absent. Facilitation of OT release to the brain by the reproductive processes, including pregnancy, parturition, and lactation, increases brain (central) OT levels. Decreased OT levels less induce maternal behavior. Additional restraint or social stressful stimuli (external stress) activate stress responses (hypothalamus-pituitary-adrenal axis, HPA) in mice. These stress signals are also activated by reproductive processes (endogenous stress). Decreased brain OT levels may not be well able to induce maternal behavior, by reducing (antagonizing) the stress response. Modified from Graphic Abstruct of Gerasimenko et al. (2021).
